# Benefits of Lactoferrin, Osteopontin and Milk Fat Globule Membranes for Infants

**DOI:** 10.3390/nu9080817

**Published:** 2017-07-28

**Authors:** Hans Demmelmair, Christine Prell, Niklas Timby, Bo Lönnerdal

**Affiliations:** 1 Dr. von Hauner Childrens Hospital, University of Munich Medical Center, D-80337 München, Germany; hans.demmelmair@med.uni-muenchen.de (H.D.); Christine.Prell@med.uni-muenchen.de (C.P.); 2 Department of Clinical Sciences, Pediatrics, Umeå University, SE-90187 Umeå, Sweden; niklas.timby@umu.se; 3 Department of Nutrition, University of California, Davis, CA 95616, USA

**Keywords:** human milk, bioactive proteins, lactoferrin, osteopontin, milk fat globule membrane

## Abstract

The provision of essential and non-essential amino acids for breast-fed infants is the major function of milk proteins. In addition, breast-fed infants might benefit from bioactivities of milk proteins, which are exhibited in the intestine during the digestive phase and by absorption of intact proteins or derived peptides. For lactoferrin, osteopontin and milk fat globule membrane proteins/lipids, which have not until recently been included in substantial amounts in infant formulas, in vitro experiments and animal models provide a convincing base of evidence for bioactivities, which contribute to the protection of the infant from pathogens, improve nutrient absorption, support the development of the immune system and provide components for optimal neurodevelopment. Technologies have become available to obtain these compounds from cow´s milk and the bovine compounds also exhibit bioactivities in humans. Randomized clinical trials with experimental infant formulas incorporating lactoferrin, osteopontin, or milk fat globule membranes have already provided some evidence for clinical benefits. This review aims to compare findings from laboratory and animal experiments with outcomes of clinical studies. There is good justification from basic science and there are promising results from clinical studies for beneficial effects of lactoferrin, osteopontin and the milk fat globule membrane complex of proteins and lipids. Further studies should ideally be adequately powered to investigate effects on clinically relevant endpoints in healthy term infants.

## 1. Introduction

Breast-feeding provides optimal support for physiological growth and development of term infants [1,2]. Microbiome, growth, body composition, prevalence of infection, and further factors, which are associated with differences in long term health outcomes [3] and later intelligence [4], differ between breast-fed and formula-fed infants. Many components, which are different between human milk and formula, are likely to be responsible for these differences. 

**Figure 1:**
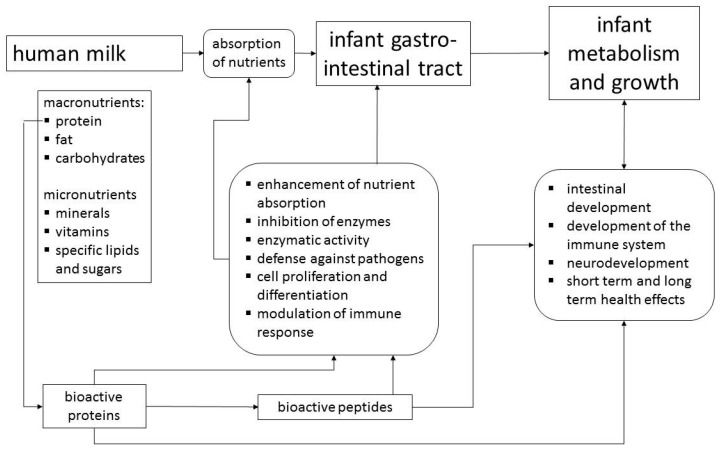
Human milk proteins support the development of the infant by their bioactivities and by providing amino acids as nutrients after digestion. Effects relate mainly to the digestive and developmental processes in the intestine, but can also be expected on whole organism level.

This review, which is based on a workshop entitled “Bioactive Proteins in Milk”, outlines the non-nutritive physiological activities of human milk components and considers in detail lactoferrin (LF), osteopontin (OPN) and milk fat globule membrane (MFGM) proteins/lipids. As the concentrations of LF and OPN in bovine milk are only about 5% and 13%, respectively, of the concentrations in human milk, although total protein content is higher, cows´ milk based formulas contain only very small amounts of these proteins [5]. While the protein and carbohydrate components of infant formulas are derived from bovine milk, the main sources of fat are usually plant oils including plant derived emulsifiers [6]. Thus, the components of MFGM with their bioactivities are not available to formula-fed infants [7]. MFGM, LF and OPN have recently become available in sufficient quantity and quality to be used as ingredients of infant formulas, which gives consideration of their functions in breast milk practical relevance. However, their functions should be viewed within the context of bioactivities of other major and minor human milk proteins (Figure 1). Proteins such as caseins, α-lactalbumin, secretory IgA and lysozyme are similarly to MFGM, LF and OPN found in human as well as bovine milk [5]. Thus, for the bioactivity in humans, species differences in the concentrations, the molecular structures, or in case of caseins and MFGM, variable compositions have to be considered [6]. On the other hand, there are proteins such as haptocorrin or bile salt stimulated lipase, which are specific for human milk. 

## 2. Human Milk Proteins with Bioactivity

Table 1 provides an overview of some milk proteins that have been investigated extensively. Bioactivities of human milk proteins, such as enhancement of nutrient absorption, enzymatic activity, modulation of intestinal microbiome, stimulation of cell proliferation, modulation of the immune system, and defense against pathogens are not limited to whey proteins, but caseins also contribute bioactivity. Table 1 indicates that described bioactivities are largely due to peptides encrypted within the proteins, which are released during digestion. Using different in vitro models of infant digestion, peptidomic analyses show that a wide variety of peptides are generated from human milk proteins, especially β-casein [8,9], while endogenous peptide concentration in human milk is between 10 and 20 mg/L [10], which corresponds to only about 0.1% of total milk protein. 

### 2.1. Casein

β-casein is highly phosphorylated, forming phosphorylated peptides during digestion, which enhance the solubility and bioavailability of calcium and zinc [11]. Furthermore, during digestion of bovine and human β-casein, peptides (β-casomorphins) are formed, which may act as opioid receptor ligands [12]. In animal experiments, oral application of β-casomorphins influences digestive motility, gastrointestinal development, and shows analgesic effects corresponding to opioidergic activity [12]. Similar effects in human infants have not been demonstrated, but β-casomorphins have been detected in the circulation of breast-fed and formula-fed infants [13]. 

κ-casein is highly glycosylated, which contributes to the stabilization of the casein micelles and with its glycosyl-residues it acts as a decoy for pathogens, as demonstrated for *H. pylori* [14]. Anti-pathogenic and bifidogenic activities of human and bovine κ-casein and its cleavage product, glycomacropeptide, are different, which might depend on the higher glycosylation and a more complex pattern of glycosyl residues in human k-casein, which also includes significant amounts of fucose [12].

### 2.2. α-Lactalbumin

The most prevalent whey protein in human milk is α-lactalbumin, which can effectively bind calcium and zinc [15,16]. Thus, peptides derived during digestion may well contribute to the absorption of divalent cations. Furthermore, proteolysis of α-lactalbumin yields peptides, which show bactericidal (characterized by a disulfide bridge), opioid agonist (corresponding to β-casomorphins, characterized by tyrosine at the N-terminus) and immunostimulating (GLF peptide) activity in vitro [12]. These bioactivities may explain why supplementation with α-lactalbumin improved the resistance of formula-fed rhesus monkeys to enteropathogenic *E. coli* induced diarrhea [17].

### 2.3. Secretory IgA and Lysozyme

Many human milk proteins have several functions besides antimicrobial activity (Table 1), but for secretory IgA and lysozyme the protection of the infant against pathogens is the only significant bioactivity. Secretory IgA provides protection to the infant from pathogens that the mother was exposed to herself. B-lymphocytes move from the maternal intestine to the mammary gland, where they are transformed to IgA producing cells [18]. An IgA dimer, paired with the secretory component, also being a glycoprotein and introducing protection against proteolysis in the gastrointestinal tract of the infant, is secreted into milk. Lysozyme, also called N-acetylmuramidase, hydrolyses peptidoglycan polymers of bacterial cell walls at the β1-4 bond between N-acetylmuramic acid and N-acetylglucosamine, thereby lysing gram positive bacteria [19]. Incubation of *Pseudomonas aeruginosa*, *Staphylococcus aureus*, and *Escherichia coli* with fresh and pasteurized human milk demonstrated bactericidal activity of fresh human milk vs. a significantly higher growth of pathogens in pasteurized milk, thus indicating heat sensitivity [20]. The significant contribution of sIgA, lysozyme and LF to the antimicrobial activity of human milk is underlined by the finding that after pasteurization, a 50–80% decrease in the immunologically detectable concentration of these proteins occurred, suggesting denaturation and loss of bioactivity.

### 2.4. Haptocorrin and Bile Salt Stimulated Lipase

As their main functions, haptocorrin and bile salt stimulated lipase (BSSL) support the absorption of vitamin B_12_ and fatty acids, respectively, by the infant. In addition, both proteins contribute to the protection of the infant against pathogens. Haptocorrin is a heavily glycosylated protein that binds and protects the acid sensitive vitamin B_12_. In vitro studies suggest that haptocorrin mediates vitamin B_12_ absorption, when the intrinsic factor system is not yet fully established [37]. Regardless of vitamin B_12_ saturation, haptocorrin inhibits the growth of enteropathogenic *E. coli* at concentrations similar to those present in human milk, which indicates that, in addition to sequestration of vitamin B_12_, further haptocorrin related mechanisms limit bacterial growth [36]. For BSSL, lipolytic activity against a wide spectrum of lipids, including cholesterol esters, lipid-soluble vitamin esters, galactolipids and ceramides has been demonstrated, whereby BSSL compensates for the limited capacity of pancreatic enzymes to digest dietary fat in early life [41]. In fact, results of a randomized clinical trial in preterm infants using a crossover design indicate that the addition of recombinant human BSSL vs. placebo to the infant feed (pasteurized human milk or infant formula) improves growth velocity and long chain polyunsaturated fatty acid absorption [42]. A subsequent multi-center study using a parallel study design enrolling 415 preterm infants confirmed a beneficial effect on growth for small-for-gestational-age infants, but not for the full study population including appropriate-for gestational age infants [48]. For mothers who produce 2-fucosylated glycans (secretor mothers), it could be shown in vitro that BSSL acts as a decoy receptor for human calicivirus strains and may provide some protection from gastroenteritis due to norovirus infection in breast-fed infants [43]. Furthermore, some isoforms of BSSL can bind to dendritic cell specific ICAM3-grabbing nonintegrin and prohibit the transfer of human immunodeficiency virus (HIV) type 1 from dendritic cells to T lymphocytes [49]. This may decrease the risk for HIV infection.

### 2.5. Lactoferrin

Human milk contains a wide variety of proteins with antimicrobial and immunomodulating activities that contribute to the protection of infants against infections and the modulation of inflammatory reactions. Some of these proteins are found in significantly higher concentrations in human milk than in bovine milk, suggesting that they are likely to be of particular importance for the development of the human infant [50]. A prominent protein in this respect is LF, a non-heme iron binding protein [51]. It is a member of the transferrin family, showing homology with serum transferrin, melanotransferrin and ovotransferrin from egg white [52]. With concentrations around 5 g/L in early milk and 2 g/L in mature milk, LF is a highly concentrated whey protein in human milk [29], but is also found in mucosal secretions such as tears, saliva, nasal and bronchial secretions [53]. LF is a 77 kDa glycosylated protein of about 700 amino acids with high homology among species. Its polypeptide chain is folded into two symmetrical lobes, called the N- and C-lobes. They are highly homologous, which suggests that they are derived from early gene duplication. The conformation is stabilized by several intramolecular disulfide bridges. Each lobe binds a Fe^3+^ ion together with a carbonate ion [54]. LF binds iron with a very high affinity (*k* = 10^−22^ mol) and to some extent also Cu^2+^, Mn^3+^ and potentially other ions [54]. With the binding of iron, the open conformation of apo-LF changes to the closed conformation of holo-LF. The conformation change is associated with increased resistance to proteolysis. The isoelectric point of LF is 8.0–8.5 and under physiological conditions it is positively charged, which is important for its functions and the interactions with membranes [53]. LF is glycosylated, but the number and location of potential glycosylation sites and those actually used vary among species. Human LF has three and cow (sheep, goat as well) LF has five potential glycosylation sites [54]. Actual usage of these sites is not well described, but only two sites are usually glycosylated in humans, and four in cow LF [55]. For human milk LF a decrease of the degree of glycosylation during the first two postnatal weeks and changes of the diversity of glycan residues during early lactation have been shown [56]. As during this period intensive changes of the gut microbiota composition occur, it is tempting to speculate about an association between these changes and LF glycosylation [56,57].

Initially research focused on the iron-binding capacity of LF, which is important with respect to absorption of iron from human milk and for bacterial protection of the infant. Most of the iron in breast milk is bound to LF, but some of the iron is associated with milk fat globules, casein and low molecular weight compounds such as citrate [58]. The molar concentration of LF in mature human milk (~25 µM) is higher than that of iron (~5 µM) and as each molecule of LF can bind two ferric ions, its iron-binding capacity is ~50 µM. Consequently, the iron saturation of human milk LF is usually below 10% and may even be as low as 3% [59,60]. This implies the possibility that non-iron saturated LF avidly can bind iron, making it unavailable for bacteria. The suppression of *E. coli* growth by human milk was convincingly demonstrated already during the 1970s [61]. Initially, sequestering of iron from bacterial pathogens was believed to be the only mechanism relevant for the antimicrobial activity of LF [62]. Only later was it demonstrated that LF can kill bacteria through an iron-independent mechanism by direct interaction of LF with the bacterial cell surface [63]. Large positively charged areas on the surface of LF facilitate direct interaction with negatively charged Lipid A, a component of the lipopolysaccharides of gram-negative bacteria [64]. This interaction can damage the bacterial membrane, altering the outer membrane permeability, resulting in the release of lipopolysaccharide [65]. In addition, by damaging the bacterial membrane, LF is able to support the antibacterial effect of lysozyme [66]. 

The bioactivity of milk LF in the gastrointestinal tract is enabled by its resistance to digestion and the comparatively low digestive capability of the infant gastrointestinal tract. Four to nine percent of the ingested LF can be detected in fecal samples of breast-fed infants [34]. Given a human milk LF concentration of about 2 g/L, a concentration of up to 200 mg/L can be estimated in the intestine [67]. This concentration has shown bacteriostatic effects in vitro and influences cell proliferation and differentiation [67]. Milk also contains sIgA, lysozyme, and lactoperoxidase and together with LF these compounds create a bacteriostatic environment in the infant intestine, similar to mucosal fluids such as tears and airway secretions [68]. Thus, bacteriostatic breast milk prohibits unrestrained bacterial growth and removes microorganisms from the small intestine without inflammation, contributing to the development of a healthy microbiome.

Milk LF does not only affect iron availability for microorganisms but also affects iron absorption. Due to the very strong iron binding affinity of LF, iron bound by LF is not available for absorption, but LF bound iron becomes available to the infant through the intestinal uptake of LF via the LF receptor, making LF a nutritional iron source with similar efficiency as inorganic iron salts [69]. LF receptors are found in a variety of adult human tissues, including salivary gland, heart, skeletal muscle, testes, adrenal gland and pancreas, but fetal LF receptors are present in high amounts only in the small intestine [70]. 

Of direct relevance for intestinal development is the finding that internalized LF influences cell proliferation and cell differentiation [71]. In Caco-2 cells, an immortalized enterocyte cell line, internalized LF influenced the extracellular signal-regulated mitogen-activated protein kinase (ERK) signaling pathway [72]. This provides a possible mechanism to explain the effect of LF on intestinal development, as the intensity of the ERK signaling pathway influences the reaction of cells at the transcription level to extracellular stimuli [73]. However, the regulatory function of LF is more complex. Although apo- and holo-LF are taken up by the receptor at similar rates, cellular signaling is more strongly activated by apo-LF [72]. Furthermore, in contrast to holo-LF, apo-LF can bind to the nucleus and act as a transcription factor [74]. Apo-LF activates the expression of proteins involved in Wnt/beta-catenin signaling, cell cycle regulation (cell proliferation), and, via TGF-β-1, cell differentiation is influenced [75]. Noteworthy, LF can interact with different immune cells, as LF receptors have been identified in lymphocytes, macrophages and dendritic cells [76]. It enhances the synthesis of signal proteins of the immune system, such as caspase 1 and IL-18, which then go into the circulation as a systemic signal [77]. 

As a maturation factor for dendritic cells, LF links innate immunity with adaptive immunity [78]. Orally administered LF has been shown to increase CD4, CD8 and NK cells in the lamina propria of the small intestine [79]. As an immunomodulator, LF can enhance and suppress immune response. The mechanistic basis of the immunomodulatory effects of LF is not yet fully elucidated, but current evidence suggests multiple mechanisms, e.g., modulation of cytokine and chemokine production, regulation of production of reactive oxygen species and immune cell recruitment [80].

These findings demonstrate that oral application of LF affects the immune system, and underlines that LF in human milk is highly relevant for the developing immune system of the infant. In utero, the infant is hardly exposed to antigens, but, after birth, the intestinal immune system has to mature rapidly, including development of tolerance to harmless antigens, as it becomes exposed to dietary and microbial antigens. This development is supported by many components, i.e., oligosaccharides, cytokines, growth factors, in breast milk [81] and LF may well be one major driver of this process. Furthermore, the findings show that effects of LF may go beyond the intestine, although most likely, the strongest effects of milk LF occur in intestinal cells. Consequently, all immunomodulatory effects demonstrated for LF so far are potentially influenced by LF in the diet, i.e., human milk or formula. 

Investigating the relevance of these effects in clinical trials depends on a sufficient supply of LF complying with all requirements for infant dietetic products. The most widely used source of LF is cow’s milk, although it contains less than one tenth of the LF content of human milk, i.e., around 0.1 g/L [5]. Most commercial methods produce pure bovine LF from skim milk or cheese whey using precipitation of other proteins by acid and ammonium sulfate, respectively, followed by purification of LF by cation-exchange chromatography [82]. Bovine and human LF are not identical, but show a 69% amino acid sequence identity, which is associated with some differences in tertiary structure [83]. However, this results in only minor differences in cellular uptake. Thus, bovine and human LF have similar functions [83]. 

As a source of human LF, several lines of transgenic dairy animals have been established, which enable the production of milk containing up to 3 g/L recombinant human LF in cow’s milk and 30 g/L in goat’s milk, respectively [84]. Furthermore, several non-animal LF expression systems employing bacteria, yeast, fungi and monocotyledon plants, have been developed [85]. Talactoferrin is a recombinant human LF expressed in the fungus *Aspergillus niger*, which shows identical amino acid sequence and similar structure to human milk LF, but differs from native human LF in the glycosylation pattern as does bovine LF [67]. An in vitro comparison of talactoferrin, bovine and human LF suggested generally similar effects [67]. To date only bovine LF has been granted GRAS (generally considered as safe) status by the US Food and Drug Administration (GRAS notice 000077). 

As LF strongly binds lipopolysaccharides, which may limit its bioactivity, contamination during purification must be avoided [83,86,87]. Another relevant aspect for the inclusion of LF into infant formulas is the iron content of the preparation, as the proportion of iron-saturated LF may unduly increase in liquid formulas, which have a considerably higher iron content than human milk [83]. During storage, iron in liquid formulas can bind to apo-LF, thus likely changing the biological effects of the supplemental LF as holo- and apo-LF affect cells differently [72]. This is of less relevance in powdered formulas, as the time span between preparation and passage through the intestinal tract is too short to allow extensive binding of iron to apo-LF [83,88].

In their 2012 review, Ochoa and colleagues identified 19 clinical studies in infants or children, which investigated potential benefits of bovine or human LF in infant nutrition [33]. The studied outcomes included iron status, anemia, fecal flora, enteric infections, immunomodulation and late onset neonatal sepsis. The authors highlight that these studies demonstrated the safety of LF, but efficacy varied between studies, with protection against enteric infection and sepsis seeming the most likely beneficial effects of LF [33]. Iron status was not affected in all studies. However, a double-blind randomized trial including 79 infants and feeding bovine LF enriched formula (850 mg/L) or standard formula (102 mg LF/L) from four weeks to 12 months of age produced two noteworthy findings [89]. In infants who completed the study, significantly less lower respiratory tract illness, and at the age of nine months significantly higher hematocrit levels were observed in the high LF group [89]. Furthermore, in a study enrolling 140 children with acute diarrhea, different oral rehydration solutions were tested. One of two rice-based solutions contained added recombinant human apo-LF and lysozyme [90]. The group receiving the recombinant proteins showed significantly shorter duration of diarrhea, a significantly higher percentage of children with solid stools within 48 h and a tendency of a lower relapse rate [90]. The positive effects cannot be ascribed to one of the active components tested but may be due to a synergy between LF and lysozyme. Nevertheless, the results agree with in vitro observations and show the potential of orally applied bioactive compounds [90].

In a recent Chinese study, effects of the addition of a small amount of bovine LF (38 mg per 100 g of powder) to otherwise identical formulas were studied in 260 infants aged 4–6 months [91,92]. Although no statistical models were applied, the results indicate that addition of LF resulted in improvements of markers of iron status (hemoglobin, serum transferrin, and soluble transferrin receptor) after the three months intervention period compared to controls [92]. Multiple linear regression analyses showed that LF addition was associated with a significantly lower incidence of respiratory and diarrhea related illnesses [91]. These results confirm the beneficial effects of LF with a surprisingly low dose of LF (i.e., less than the concentration present in cow milk), although it is not clear how applicable they are to other formula compositions and different socioeconomic settings. 

The Ochoa review also included a study in preterm infants. In a double-blind randomized trial it was tested whether the risk of late-onset sepsis, a very serious health risk for preterm infants, could be reduced by oral administration of bovine LF (100 mg per day) alone or in combination with a probiotic *Lactobacillus* strain compared to a glucose control solution [93]. Application of the study products started on day three of life and infants (*n* = 472) were routinely fed breast milk or preterm formula. The incidence of an episode of late onset sepsis was significantly lower in the LF groups (5.9% and 4.6% of infants, respectively) compared to 17.3% in the placebo group [93]. Considering the importance of nutritional support to reduce the risk of sepsis and necrotizing enterocolitis (NEC) and the potential of LF indicated by this first study, further studies in preterm infants have been or are being currently performed. Based on the already completed studies, a 2015 Cochrane review concludes that available evidence suggests oral bovine LF decreases late onset sepsis and NEC stage II or greater in preterm infants without adverse effects [94]. Although the evidence is still considered weak and results of ongoing trials have to be awaited [94], dietary supplementation of preterm infants may well soon be a clinical application of LF in infant nutrition and translate an enormous amount of laboratory and clinical research into health care practice. 

### 2.6. Osteopontin

LF has received much attention while searching for ways to improve infant formula by including bioactive milk components. Although it contributes about 2% of total human milk protein, OPN has only recently been considered as a potential formula component [45]. Similar to LF, OPN concentrations are much lower in bovine milk (approximately 18 mg/L) than in human milk, which results in even lower OPN concentrations of approximately 9 mg/L in infant formulas [45]. Nevertheless, bovine OPN can be isolated from cow’s milk by ion exchange chromatography in sufficient quantity and quality to be applied in clinical testing [95].

OPN, previously called Eta-1 (early T-lymphocyte activation-1) or SSP1 (secreted phosphoprotein 1), was initially identified as a linking protein and crucial factor in extracellular bone biomineralization [96]. OPN is an acidic, thus at physiological pH negatively charged, glycosylated, and highly phosphorylated protein [97]. It is one of several small integrin-binding ligand, N-linked glycoproteins (SIBLING proteins) mediating cell-matrix interactions and cell signaling [98]. OPN is encoded by a single gene, but a series of isoforms with molecular weights from 41 to 75 kDa result from alternative splicing, alternative translation and different post-translational modifications [99]. Thus far, three splice variants of the human OPN transcript have been identified: OPN_a_, the full-length isoform; OPN_b_, which lacks exon 5, and OPN_c_, which lacks exon 4 [100]. Although an intracellular OPN (OPN_i_) recently was described, OPN is primarily a secreted protein involved in a series of physiological and pathophysiological processes.

OPN is expressed in a variety of cell types and tissues including pre-osteoblasts, osteoblasts, osteocytes, dendritic cells, macrophages and T cells, hepatocytes, skeletal muscle, endothelial cells, brain, and mammary gland [99]. Extracellular OPN functions through its interactions with cell surface integrins and the CD44 receptor. It influences biomineralization, tissue remodeling and immune regulation [99]. OPN is significantly involved in regulation of T cell development, supporting establishment of the T helper (Th) 1 pathway, suppressing Th2 cells [101], and under certain conditions may stimulate IL-17 secretion [102]. Evidence suggests that OPN plays a critical role in autoimmune diseases, in several types of cancer [103] and cardiovascular disease [104]. A further important function relates to the involvement of OPN in the regulation of myelination in the central nervous system [105]. 

As expected from the expression of OPN in many different cells, it is found in biofluids such as plasma, urine and milk. Compared to the low concentration in adult plasma (~35 µg/L) the concentration in human milk is high, but variable among mothers (138 ± 79 mg/L, mean ± SD) [45]. Although the concentration of OPN is low in colostrum, high levels are established after three days of lactation. The levels decrease with advancing lactation, but about half maximal levels are maintained beyond 1 year of lactation [106]. The number of macrophages in milk decreases with duration of lactation, suggesting that mammary epithelial cells are a more important source of milk OPN than macrophages [106]. In human and bovine milk only the full-length isoform OPN_a_ has been detected [95]. OPN fragments found in both milks are due to proteolytic activity in milk. Although the chain length of human and bovine OPN is different with 298 amino acids in human OPN compared to 262 amino acids in bovine OPN, there is a 61% sequence homology, and sites important for post-translational modifications, proteolytic cleavage and integrin-binding are similar [95]. The OPNs share the integrin-binding Arg-Gly-Asp (RGD) motif and there is a high similarity in a further motif with Ser-Val-Val-Tyr-Gly-Leu-Arg (SVVYGLR) in the human and Ser-Val-Ala-Tyr-Gly-Leu-Lys in the bovine form [97]. Human milk OPN typically has 25 phosphate residues (mainly bound to serine), whereas bovine OPN usually contains 22 phosphates. There is also a similarity of glycosylation sites, but different carbohydrate moieties are attached [95]. 

The functions of OPN, including the role of human milk OPN in the development of immunological functions and nervous tissue, are not yet fully understood. However, the high concentrations of OPN in human milk and cord blood indicate an importance of OPN in lactogenesis and/or infant development and programming of long-term health outcomes [45,107]. In vitro experiments have indicated that human and bovine milk OPN are in part resistant to proteolysis in the infant intestinal tract, which makes OPN a potentially bioactive component of human milk [108]. In animal studies after oral application of OPN some proteolytic cleavage products or even intact OPN could be detected in plasma using antibodies raised against OPN [109,110] and these peptides show biological effects [110]. 

As only the highly phosphorylated full length isoform of OPN is found in milk [95,97], milk OPN is substantially different from OPN forms implicated in the disorders mentioned above [95]. However, considering the various roles of OPN in physiological and pathological processes, investigation of its safety is an important issue. In this context, it is reassuring that in vitro studies did not identify any genotoxic or cytotoxic effects, and a 13 week oral toxicity test in rats with a diet containing up to 2% Lacprodan(R) OPN10 (about 80% pure bovine OPN) did not show any clinically relevant adverse effects related to this product [111].

For assessment of the safety and potential efficacy of bovine OPN supplementation, Donovan et al. compared growth, body composition, bone mineral density, hematological measures and intestinal mRNA expression, applying a microarray hybridization technique, in infant rhesus monkeys, which are a good model to test infant formulas [46]. Monkeys were either fed standard formula or formula enriched with 125 mg bovine OPN per liter (*n* = 6/group) from birth until the age of three months. Breast-fed infant monkeys (*n* = 4) were included as a reference. Anthropometry and hematology revealed no significant differences between the two formula-fed groups, which indicates safety of the tested OPN dosage [46]. The potential of bovine OPN to influence intestinal development was shown by the fact that adding OPN to the formula made gene expression of close to 2000 genes more similar to gene expression in breast-fed infants. OPN influenced genes related to cell cycle progress (e.g., CUX1), cell-cell communication, cell movement and cell survival (e.g., EGFR) and regulators of foregut development (FOX genes). The potential importance of dietary OPN is supported by the fact that many of the identified genes are related to pathways which have previously been found to be influenced by OPN via its binding to integrins and CD44. [46]. These findings suggested that formula-fed infants might benefit from OPN supplementation.

### 2.7. Clinical Trial with OPN

In a recent randomized, double-blinded clinical study, 240 infants were fed a whey predominant standard formula or the same formula with addition of 65 or 130 mg bovine OPN per liter [47]. As an additional non-randomized control group, 80 breast-fed infants were included. Formulas were fed between one and six months of age, and examinations including blood sampling were performed at the ages of one, four and six months. The main study outcomes were growth, health, nutritional status and cytokine expression. OPN containing formulas were well tolerated, and no significant differences in formula intake, growth or iron status were found among formula groups. Differences between breast-fed and formula-fed infants were consistent with previous findings. The important clinical outcome of the study was the lower incidence of fever in both OPN supplemented groups (4.0% ± 7.8%, 5.5% ± 10.1%, respectively) compared to the standard formula group (8.2% ± 11.7%) during the intervention period. In fact, the OPN groups were not significantly different from the breast-fed group (3.2% ± 7.3%). The plasma cytokine patterns revealed differences between breast-fed and formula-fed infants, but also differences among formula groups, supporting beneficial effects of oral OPN supplementation. Both OPN supplemented groups showed lower levels of pro-inflammatory TNF-α and higher levels of Interleukin-2 compared to the standard formula group at four months of age [47]. As higher TNF-α levels in formula-fed compared to breast-fed infants have been interpreted as a pro-inflammatory immune response to early formula feeding [112], this indicates that OPN might beneficially affect the development of the immune system.

These initial findings of benefits of dietary OPN in infants and animal models support the concept that OPN is a bioactive component in human milk, whose inclusion might improve infant formulas. Further experiments and clinical studies are warranted to improve the understanding of the functions of dietary OPN and to confirm safety and efficacy of formulas enriched with bovine OPN. 

### 2.8. Milk Fat Globule Membrane

Milk fat globules are surrounded by a triple membrane system with the inner monolayer derived from the endoplasmatic reticulum of the mammary cells and the outer bilayer derived from the apical membrane of epithelial cells of the lactating mammary gland. The MFGM is a complex construct containing many cellular components including cholesterol, glycerophospholipids, sphingolipids and proteins [113]. The hydrophilic outside of the membrane prohibits coalescence of the globules and establishes a stable oil-in-water emulsion. The MFGM is about 10–20 nm thick and accounts for 2–6% of the globule mass [114]. As the MFGM is derived from the apical membrane, the endoplasmic reticulum and other intracellular compartments of the mammary epithelial cell, identified proteins reflect their cellular origin or their involvement in lipid synthesis and formation of lipid droplets [115]. Noteworthy, for many MFGM proteins, bioactivities have been shown or suggested. Among well described proteins present in high concentrations in the MFGM are Mucin 1 (MUC 1, PAS 0), xanthine oxidoreductase (XDH/XO or XOR), butyrophilin (BTN), lactadherin (PAS 6/7, MFG-E8), CD 36, adipophilin, and fatty acid-binding protein [116]. For some of the proteins gastric stability has been demonstrated [117] and they have been suggested to contribute to the protection against bacteria and viruses in the neonatal gastrointestinal tract and to affect the immune system. 

Due to its variable number of tandemly repeated amino acid sequences with high threonine and serine content, the molecular weight of MUC1 varies between 250 and 450 kDa [118]. MUC1 is an integral membrane protein with a heavily glycosylated extracellular domain, which is found in most epithelial tissues [118]. In vitro, MUC1 inhibits the invasion of Caco-2 and FHs74 cells (a model of fetal intestinal cells) by *Salmonella typhimurium* at concentrations similar to human milk concentrations [119]. Furthermore, MUC1 has been shown to bind to intercellular adhesion molecule-3-grabbing non-integrin (DC-SIGN) of dendritic cells, which prohibits the transmission of HIV from dendritic cells to T cells and thus may inhibit the transmission of HIV from the mother to the infant via breast-feeding [120]. Via interaction with DC-SIGN, expressed by dendritic cells in the infant gastrointestinal tract, MUC1 blocks the interaction of pathogens with dendritic cells and may well contribute to shaping infant immunity [121]. 

XOR is a dimeric metalloflavoprotein made up of two subunits of 145 kDa each, including a molybdenum and a flavin-adenine-dinucleotide containing cofactor [122]. Acting as an oxidase, XOR transfers redox equivalents to molecular oxygen leading to the generation of cytotoxic reactive oxygen species (hydrogen peroxide, superoxide anion, hydroxyl radicals). Furthermore, XOR can reduce nitrite to nitric oxide, which together with superoxide anions gives rise to reactive nitrogen species [123]. Antimicrobial activity of XOR may result from providing hydrogen peroxide for the antibacterial activity of lactoperoxidase and more directly from reactive nitrogen species, which may be derived from nitrite secreted by enteric bacteria [123]. An ability of endogenous breast milk XOR to generate nitric oxide and to attenuate the growth of *Escherichia coli* and *Salmonella enteritides* has been shown in vitro [124]. 

An MFGM protein with antiviral activity is lactadherin, a glycosylated, membrane associated protein with a molecular mass of approximately 46 kDa [118]. Its amino acid sequence contains an RGD (Arg-Gly-Asp) motif, which enables binding to integrins, and a sequence with basic amino acids, which enables binding to membrane phosphatidylserine [118]. Lactadherin, isolated from human milk, prevented the replication of rotavirus in tissue culture [125]. The pre-incubation of rotavirus infected cells with lactadherin, isolated from human milk, prior to feeding the cells to mice reduced the occurrence of experimental gastroenteritis by more than 90% compared to feeding infected cells pre-incubated with lactadherin-devoid infant formula [125]. Although the mechanism behind the antiviral effect of lactadherin could not be clarified, the strong reduction in activity after acidic hydrolysis of sialic acid from the protein points towards the importance of glycosylation. An observational study of human infants during the first 6 months of life found that the occurrence of symptomatic rotavirus infection was negatively associated with the amount of human milk lactadherin consumed, while intake of mucin and secretory IgA with milk was unrelated [126]. Furthermore, lactadherin might contribute to establishing the intestinal barrier in the neonate [127]. Mice depleted of lactadherin (=MFG-E8) by either administrating an MFG-E8 antibody or specific deletion of the MFG-E8 gene, showed slower enterocyte migration along the crypt-villus axis after mucosal injury [128], while intraperitoneal application of recombinant MFG-E8 restored enterocyte migration. MFG-E8 is expressed by macrophages of the murine small intestine [128] and it may be assumed that milk MFG-E8 supports growth and maintenance of the epithelia, as it has been identified as a factor that links apoptotic cells to phagocytes [129]. Lactadherin has been found to be involved in a series of further processes, including angiogenesis and stimulation of the immune system, and some of these effects could well apply for MFGM lactadherin [130]. 

Another quantitatively major protein in human MFGM is butyrophilin, which was initially identified in cow’s milk with a molecular mass of about 66 kDa, showing only N-glycosylation. It was soon recognized as a member of the Ig superfamily of proteins [116]. Widespread low level expression of butyrophilin variants has been shown in humans and human milk butyrophilin was renamed BTN1A1. The butyrophilins (e.g., BTNA2A) modulate T cell responses upon antigen presentation [131]. Investigations in BTN1A1 knockout mice showed that, without BTN1A1, secretion of milk fat globules was severely compromised, limiting survival of the offspring [132]. It has been shown in mice that recombinant BTN1A1 interacts with T cells as does BTN2A2 and inhibits T cell metabolism [133]. Considering the relevance of butyrophilins for the immune system, BTN1A1 might be another human milk protein supporting development of the infant immune system.

Detailed proteomic studies, using matrix assisted laser desorption mass spectrometry, have identified a large variety of further proteins in the MFGM of human milk present in lower concentrations. Liao et al identified 191 proteins and grouped them according to allocated functions. Almost 20% of the proteins were related to immune response and another 19% to cell communication/signal transduction [115], which indicates potentially many further bioactivities. As the proteome of bovine MFGM has been found similar to the human MFGM proteome [134], these findings suggest the possibility of beneficial effects of supplementing infant formulas with bovine MFGM. 

On a per weight basis, 25–70% of MFGM are proteins [113]. In addition, MFGM contributes further membrane components to the diet of breast-fed infants. A major class of compounds are sphingolipids, which are based on a sphingosine backbone and according to the head group can be differentiated into sphingomyelin (phosphocholine), ceramide (H), glycosylceramides (glucose or lactose), lactosylceramides (lactose) and with more complex glycosyl residues gangliosides (monosaccharides, N-acetylgalactoseamine, sialic acid and others) [135]. A series of biological functions have been ascribed to ceramides, including regulation of cell growth, apoptosis and inflammation, but cellular uptake seems limited [136]. Similarly, intact sphingomyelin is not absorbed, but it accelerates maturation of the intestine of rat pups [137]. By the activity of alkaline sphingomyelinase and ceramidase, which are expressed on the apical membranes of intestine epithelial cells, absorbable sphingosine is derived from sphingomyelin, which can be converted to spingosine-1-phosphate by sphingosine kinase [136]. Sphingosine has been found to induce cell cycle arrest and apoptosis and to inhibit protein kinase c [138]. In the intestine, sphingosine-1-phosphate seems to play an important regulatory role for immune functions [139]. Further, rat studies suggest systemic effects of dietary sphingomyelin, including an increase of nervous system myelination in a deficit model [140,141]. With regard to neurodevelopment, the ganglioside content of the MFGM might be highly relevant, considering the high ganglioside content in nervous tissue, the high requirement in the perinatal period due to the rapid brain growth, and the demonstrated uptake of dietary gangliosides [142]. Furthermore, gangliosides, which are exclusively located in the MFGM, are important as they act as decoy receptors for pathogens, which may prevent infections of infants, and gangliosides are able to modulate the behavior of immune cells [143]. MFGM concentrate, in combination with LF, has been shown to influence the microbiome of piglets and gangliosides may well contribute to this observation [144]. In agreement with this, in infant rats the addition of bovine MFGM, whose lipids differ from human milk MFGM, to the formula made intestinal development and microbiome of formula fed rats more similar to that of breastfed rats [6,145]. In addition, MFGM contributes significant amounts of choline [146], cholesterol [113], and sialic acid via glycosylated proteins and lipid-bound sialic acid from gangliosides [143,147] to the dietary intake of infants. There is considerable evidence from in vitro studies, animal studies and observational studies, that these compounds are linked to infant development, including development of cognitive functions [148,149,150]. 

### 2.9. Clinical Trials with MFGM Components

Considering the huge variety of identified specific functions of MFGM lipids and proteins, it seems well justified to test MFGM in clinical studies on infants [7]. In fact, in recent years several randomized and double-blinded trials have studied the effects of adding MFGM to infant diets on health and cognitive function. In 6–12-month-old infants in Peru, daily supplementation with MFGM enriched protein significantly decreased the duration of diarrhea episodes and the incidence of bloody diarrhea by almost 50% considering confounding factors [151]. In agreement with these findings, in 2.5–6-year-old European preschool children a daily milk-based supplement with MFGM phospholipids reduced the number of days with fever during the four-month intervention period significantly compared to a corresponding supplement without MFGM components [152]. A trial in India, testing for protection against diarrhea by supplementation with a ganglioside concentrate during the second year of life, showed acceptance and safety of the product, but was inconclusive in respect to efficacy [153]. A large non-inferiority study in France and Italy found no differences of growth between infants given two MFGM preparations, which differed in their protein to lipid ratio, compared to standard formula from two weeks to four months of age [154]. The MFGM formulas were generally well tolerated (no group differences in parental reports of vomiting, fussing, crying, colitis), but post-hoc statistical analysis indicated a higher incidence of eczema in the group receiving the high protein MFGM preparation [154]. It is of interest to note that a Swedish study (described below) tested the same protein-enriched MFGM preparation as the French/Italian study, but did not observe any sign of increased incidence of skin reactions in the supplemented group compared to standard formula or breast-feeding [155].

In a Swedish study, term infants were randomized before the age of two months to a formula supplemented with a protein-rich MFGM preparation (4% of total protein) or a standard formula [156,157,158]. The formulas were fed until the age of 6 months and the infants were followed until the age of 12 months together with a breast-fed reference group. At 12 months of age, follow-up data could be collected from 73 infants of the MFGM group, from 68 infants in the standard formula group and from 72 breast-fed infants. Some of the observed effects seem related to difference in energy density and macronutrient composition between the study formulas, but some important observations are most likely related to the bioactivity of MFGM components. During the period of study formula feeding, the MFGM supplemented group had a significantly lower incidence of acute otitis media than the standard formula group (1% vs. 9%), and lower incidence (25% vs. 43%) of antipyretic use [158], which agrees with previous findings. In addition, the observed lower levels of IgG antibodies against *pneumococci* after vaccination in the MFGM group compared to the standard formula group agree with an immune modulatory effect of MFGM components. Among the primary outcomes of the study was the assessment of neurodevelopment using Bayley Scales of Infant and Toddler Development at the age 12 months [156]. While scores of the motor and verbal domains were not different between the randomized groups, the MFGM supplemented group obtained significantly higher scores in the cognitive domain compared to the standard formula group (105.8 ± 9.2 vs. 101.8 ± 8.0, M ± SD), which did not differ from the breast-fed group (106.4 ± 9.5). This is in line with findings in infants in Indonesia, who received standard formula or the same formula supplemented with complex milk lipids from 2 to 24 weeks of age [159]. After the intervention period, serum ganglioside concentrations were significantly higher in supplemented infants, who scored significantly better in hand-eye coordination IQ and performance IQ determined by use of the Griffiths Mental Development Scale [159]. 

Available evidence from model studies and clinical trials indicates the potential of beneficial effects of the combination of bioactive compounds or any specific component of MFGM to improve infant formulas. Importantly this relates to actual health during infancy and may contribute to optimizing the long term programming of the immune system and cognitive functions.

## 3. Conclusions

The bioactivities of human milk components in the infant intestine and on a systemic level are in many cases not provided by current formula milk components. This difference may in part explain the advantage of breast-feeding in respect to short term effects on infant health, e.g., incidence of infectious diseases, and long term outcomes, such as the risk for obesity, diabetes, cardiovascular disease, and cognitive performance compared to formula-feeding. The availability of some corresponding components from bovine milk or biotechnological production offers the possibility to include these components into formulas and to further close the gap between formula-feeding and breast-feeding. LF has been tested in a large number of studies and also MFGM or some of its components have been applied in various research settings, while, in the case of OPN, only initial trials have been performed so far. It is of importance to note that at least some of the beneficial effects of LF, OPN and MFGM could be due to synergies between these compounds or additional human milk components, and thus may not manifest after addition of isolated components to infant formulas. Model experiments in vitro and in animals may help to define required complex mixtures. Nevertheless, in order to gain solid evidence and quantitative estimates of the benefits for different groups of infants, adequately powered, well designed randomized, controlled trials with optimized ingredient mixtures are required. These studies shall also provide further evidence for the safety of LF, OPN and MFGM. 

## Figures and Tables

**Table 1:** Overview of proteins and peptides in mature human milk with presumed functions or activities in addition to being a source of amino acids to breast fed infants.

Protein	Human Milk Concentration (mg/L)	Molecular Weight (kDa)	Human Milk Concentration (µmol/L)	Bioactivity ^a^	Bioactivity in Cell or Animal Models (1) or in Clinical Trials (2)	Resistance to Digestion
β-casein	3000–5000 [21]	24	125–208	peptides	(1) casein phosphopeptides support Ca absorption [11], (1) β-casomorphins act as opioid receptor ligands [22] (2) β-casomorphins appear in infant plasma [23]	casein phosphopeptides detected in ileostomy fluids of adults [24]
κ-casein	1000–3000 [21]	30	33–100	protein/peptides	(1) inhibition of *H. pylori* adhesion to gastric mucosa cells [14] (1) glycomacropeptide shows bifidogenic and pathogen inhibiting effects in the gut and anti-inflammatory effects after absorption [12]	κ-caseinglycopeptide detected in the duodenum of adults after milk consumption [25]
α-lactalbumin	1800–3100 [26]	14	129–221	peptides	(1) activity against gram positive bacteria [13] (1) immunostimulation by GLF peptides [27] (2) increased serum Fe [28]	slower digested than caseins, but no intact α-lactalbumin detected in infant faecal samples [27]
lactoferrin	1200–3000 [29]	77	16–39	protein/peptides	(1) antimicrobial and immunomodulating effects, influence on iron absorption in various models [30] (1) antimicrobial activity of lactoferricin H and B [31] (1) bifidogenic activity [32] (2) beneficial effects [33]	detected in fecal samples of breast fed infants [34]
haptocorrin	<0.7–7	68	<0.01–0.1 (age 4 months) [35]	protein	(1) bacteriostatic effect of porcine haptocorrin [36] (1) contribution to Vit-B12 absorption [37]	in vitro resistance to proteolysis for porcine haptocorrin [36]
lysozyme	50–250 [38]	15	3–17	protein	(1) cleavage of cell wall of gram positive bacteria [19]	resistant to peptic digestion, but susceptible to tryptic digestion [39]
secretory IgA	500–1000 [38]	420	1–2	protein	(1) antibody, antimicrobial activity [18]	secretory IgA detected in fecal samples of breast fed infants [34]
bile-salt stimulated lipase	100–200 [40]	120–140	<1–2	protein	(1) lipolytic activity [41] (2) increased long chain fatty acid absorption [42] (1) inhibits the attachment of Norwalk virus-like particles to its cellular ligand [43]	stable at pH > 3 [44]
osteopontin	60–220 [45]	60	1–4	protein/peptides	(1) altered intestinal gene expression in rhesus monkeys [46] (2) less fever and altered cytokine pattern [47]	human and bovine osteopontin are partially resistant to proteolysis by infant gastric juice at pH 4 [41]

^a^ Bioactivity ascribed to intact protein or derived peptides.
